# Computational search for hypotheses concerning the endocannabinoid contribution to the extinction of fear conditioning

**DOI:** 10.3389/fncom.2013.00074

**Published:** 2013-06-03

**Authors:** Thomas J. Anastasio

**Affiliations:** Computational Neurobiology Laboratory, Department of Molecular and Integrative Physiology, Beckman Institute, University of Illinois at Urbana-ChampaignUrbana, IL, USA

**Keywords:** extinction of fear conditioning, endocannabinoids, neural networks, formal methods, declarative programming, state-space search

## Abstract

Fear conditioning, in which a cue is conditioned to elicit a fear response, and extinction, in which a previously conditioned cue no longer elicits a fear response, depend on neural plasticity occurring within the amygdala. Projection neurons in the basolateral amygdala (BLA) learn to respond to the cue during fear conditioning, and they mediate fear responding by transferring cue signals to the output stage of the amygdala. Some BLA projection neurons retain their cue responses after extinction. Recent work shows that activation of the endocannabinoid system is necessary for extinction, and it leads to long-term depression (LTD) of the GABAergic synapses that inhibitory interneurons make onto BLA projection neurons. Such GABAergic LTD would enhance the responses of the BLA projection neurons that mediate fear responding, so it would seem to oppose, rather than promote, extinction. To address this paradox, a computational analysis of two well-known conceptual models of amygdaloid plasticity was undertaken. The analysis employed exhaustive state-space search conducted within a declarative programming environment. The analysis reveals that GABAergic LTD actually increases the number of synaptic strength configurations that achieve extinction while preserving the cue responses of some BLA projection neurons in both models. The results suggest that GABAergic LTD helps the amygdala retain cue memory during extinction even as the amygdala learns to suppress the previously conditioned response. The analysis also reveals which features of both models are essential for their ability to achieve extinction with some cue memory preservation, and suggests experimental tests of those features.

## Introduction

Neurobiologically, fear conditioning and its extinction are better understood than any other forms of emotional learning (Maren, 1999; Ledoux, 2000; Herry et al., 2010). Fear conditioning can be accomplished by pairing an initially neutral cue stimulus with a normally aversive fear-inducing stimulus. A successfully conditioned cue elicits the fear response when presented alone. Subsequently, repeated presentation of the cue alone can cause extinction. An extinguished cue no longer elicits the fear response. The processes of fear conditioning and extinction are thought to engage some of the same neural mechanisms that are implicated in fear and anxiety disorders, and the hope is widely shared that better understanding of the neurobiology of fear conditioning and extinction will translate into better treatments for mental illnesses such as post-traumatic stress disorder (PTSD; Mahan and Ressler, 2012).

Although several brain regions are involved in these processes, fear conditioning and extinction depend most heavily on synaptic plasticity occurring within the amygdala (Blair et al., 2001). Recent work suggests that the endocannabinoid system also plays a role. The main endocannabinoid receptor in the brain is CB1 (Piomelli, 2003), and CB1 is highly expressed in several amygdaloid nuclei (McDonald and Mascagni, 2001). Seminal research demonstrated that mice deficient in CB1 have normal fear conditioning but impaired extinction (Marsicano et al., 2002). Follow up studies confirmed that suppression of cannabinoid signaling impairs extinction (Holter et al., 2005; Arenos et al., 2006; Kamprath et al., 2006; Niyuhire et al., 2007; Ganon-Elazar and Akirav, 2009) while enhancement of cannabinoid signaling facilitates extinction (Chhatwal et al., 2005; Bitencourt et al., 2008).

The effect of CB1 activation is to cause long-term depression (LTD) of the GABAergic synapses of inhibitory interneurons onto certain amygdaloid projection neurons (Marsicano et al., 2002). Some of these projection neurons are likely to be the same ones that transfer conditioned cue signals from the input stage to the output stage of the amygdala. Because CB1-mediated LTD would enhance their cue responses it would seem to oppose, rather than promote, extinction. Computational analysis was undertaken to address this apparent paradox. The analysis was based on two established conceptual models of amygdaloid plasticity (Pare et al., 2004; Lafenetre et al., 2007).

The two models were specified as computer programs written in a declarative programming language, which allowed exhaustive search of their state spaces. Imperative programming languages, which are more conventional, are not conducive to such an analysis. The declarative approach was used to determine how many synaptic strength configurations in each model are jointly compatible with CB1-mediated GABAergic LTD, with extinction of fear conditioning, and with a range of constraints derived from experimental findings. Owing to the uncertainty that still surrounds the actual adaptive mechanisms responsible for extinction, it can be assumed that the likelihood of any learned outcome is proportional to the number of synaptic strength configurations that are compatible with that outcome. The results lead to new insights into the possible contribution of CB1-mediated LTD to the extinction of fear conditioning, and suggest effective ways in which each model could be tested experimentally.

## Methods

The methods applied here begin with two well-known conceptual models that offer explanations for how fear conditioning, and extinction of fear conditioning, could be produced by synaptic plasticity occurring within the amygdala. Both conceptual models adhere closely to known amygdaloid anatomy and physiology. These two conceptual models are translated into computational models (i.e., they are implemented as computer programs), henceforth referred to as Model 1 and Model 2, which are relatively simple but capture the basic features of amygdaloid connectivity and plasticity. Their relative simplicity enables them to be analyzed using exhaustive searches of their state spaces.

The neural elements in both Model 1 and Model 2 can be interpreted either as single neurons, or as groups of neurons within the same subregion that all have the same connectivity and behavior (see Discussion). The responses of the neural elements in the models depend entirely on the weights of the connections between them, so the state of either model is completely determined by the configuration (combination) of its connection weights (representing synaptic strengths). As these weights change, according to rules of plasticity, the state changes, and model analysis involves searching the space of all possible states (i.e., all possible connection weight combinations) for configurations that produce fear conditioning followed by extinction but that also conform to certain constraints as determined by experimental findings. The analysis of each model begins with minimal constraints. Subsequent searches with progressively more restrictive constraints reveal the possible role of the endocannabinoid system in extinction of fear conditioning, and also reveal which features of each model are the most critical for the performance of that role.

### Amygdaloid connectivity

**Figure 1:**
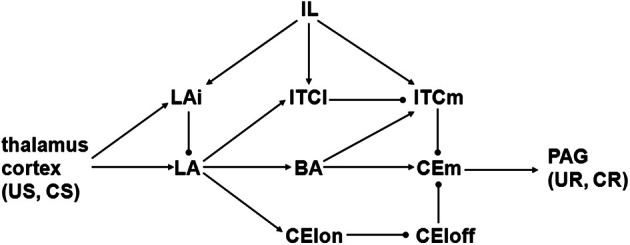
**Schematic diagram illustrating connectivity of amygdaloid nuclei and cell groups.** Arrow and ball connections are excitatory and inhibitory, respectively. BA, basal nucleus; CElon and CEloff, “on” and “off” cell groups in lateral part of central nucleus; CEm, medial part of central nucleus; CR, conditioned response; CS, conditioned stimulus; IL, infralimbic part of prefrontal cortex; ITCl, ITCm, lateral and medial intercalated cell masses; LA, lateral nucleus; LAi, interneurons in lateral nucleus; PAG, periaqueductal gray; UR, unconditioned response; US, unconditioned stimulus.

A schematic illustrating the known synaptic connectivity within the amygdala, and between the amygdala and certain other brain regions, is shown in Figure 1. The basic scheme proposed by Ledoux (2000) is that signals related to unconditioned stimuli (US, usually pain) and conditioned stimuli (CS, usually sound) converge on the lateral nucleus of the amygdala (LA), and are relayed from there via other amygdaloid nuclei to the medial part (CEm) of the central nucleus (CE), which then projects to the periaqueductal gray (PAG) and other pre-motor structures that mediate unconditioned and conditioned (UR and CR) fear responses. A recent review proposes the following set of specific interconnections (Pare et al., 2004). Sensory signals driven by US and CS arrive at LA from thalamus and cortex. LA activates the basal nucleus of the amygdala (BA), and BA activates CEm, so LA could active CEm via BA. LA could also relay US and CS signals to CEm via the inhibitory intercalated cell masses (ITCs) in the amygdala. The ITCs can be roughly divided in two, where the lateral (ITCl) inhibits the medial (ITCm) mass, and ITCm inhibits CEm. ITCl receives excitatory input from LA, so LA can also activate CEm via disinhibition: LA excites ITCl and ITCl inhibits ITCm, thereby releasing CEm from ITCm inhibition. Note that BA can also inhibit CEm via ITCm.

The forgoing scheme is based on the following anatomical and physiological findings from mammals, mainly rodents. Thalamic nuclei including the medial geniculate and the posterior intralaminar project to LA (Turner and Herkenham, 1991; Linke et al., 2000). Insular, temporal, and other cortices also project to LA (McDonald, 1998). These thalamic and cortical, glutamatergic projections convey both US and CS signals to LA. They also arrive at CEm but those connections have been omitted from the schematic (see Results). Small to medium size GABAergic interneurons in LA (LAi in Figure 1) also receive thalamic and cortical inputs and inhibit glutamatergic projection neurons in LA (McDonald, 1985; Woodson et al., 2000; Shumyatsky et al., 2002). LA projects to BA (Pitkanen and Amaral, 1991; Pitkanen et al., 1997), and LA projects to the lateral part of the central nucleus (CEl) but not to CEm, while BA projects to CEm but not to CEl (Krettek and Price, 1978; Pare et al., 1995; Pitkanen et al., 1997). CEm projects to PAG (Rizvi et al., 1991; Da Costa Gomez and Behbehani, 1995).

LA and BA send glutamatergic projections to the ITCs, with LA and BA preferentially contacting ITCl and ITCm, respectively (Royer et al., 1999). ITCl sends a GABAergic projection to ITCm and ITCm, in turn, sends a GABAergic projection to CEm (Pare and Smith, 1993; Royer et al., 1999, 2000). Thus, LA disinhibits CEm by exciting ITCl, which inhibits ITCm. A similar disinhibitory pathway involving GABAergic interneurons in CEl has been identified. CElon and CEloff neurons are respectively excited and inhibited by the CS, and CElon responses precede CEloff responses, suggesting that CElon inhibits CEloff (Ciocchi et al., 2010). Both types project to CEm. Since LA projects to CEl it is thought that LA can also disinhibit CEm by exciting CElon, which inhibits CEloff. One study suggests that CEl is necessary for fear conditioning (Ciocchi et al., 2010), while another suggests that CEl is mainly involved in the conditioned inhibition produced when a CS is unpaired with a US (Amano et al., 2010). Due to this uncertainty, the CEl pathway is included in neither model but, since the ITC and CEl pathways have the same form, some results on the ITC disinhibitory pathway could apply as well to the CEl disinhibitory pathway.

The infralimbic (IL) part of the prefrontal cortex projects to several amygdaloid nuclei including LA and both ITCl and ITCm (Hurley et al., 1991; McDonald et al., 1996). IL neurons respond to the CS after the fear response to it has been extinguished (Milad and Quirk, 2002; Milad et al., 2004), and IL can suppress the responses of CEm neurons (Quirk et al., 2003), suggesting that IL participates in extinction memory and expression. Two possible pathways by which IL could suppress CEm have been explored physiologically. One group finds that IL activates mainly inhibitory interneurons in LA, and this can suppress the activation of LA projection neurons (Rosenkranz and Grace, 2001, 2002; Rosenkranz et al., 2003). Another group finds that IL can suppress CEm responses even when CEm is activated by direct stimulation of LA (Quirk et al., 2003). In this case it is likely that IL is inhibiting CEm via ITCs (Likhtik et al., 2008).

### Amygdaloid plasticity

Fear conditioning is associated with synaptic plasticity occurring within the amygdala (for review see Maren, 1999). Many studies demonstrate long-term potentiation (LTP) of thalamic and cortical input fiber synapses onto LA neurons (Chapman et al., 1990; Rogan and Ledoux, 1995; McKernan and Shinnick-Gallagher, 1997; Huang and Kandel, 1998; Bauer et al., 2002; Bauer and Ledoux, 2004). High-frequency stimulation of thalamic and cortical inputs causes LTP of those input fiber synapses both onto excitatory projection neurons (LA in Figure 1) and onto inhibitory interneurons (LAi), and also causes LTP of the synapses of LAi onto LA neurons (Mahanty and Sah, 1998; Bauer and Ledoux, 2004). High-frequency stimulation of LA produces LTP of the synapses of LA projection neurons onto BA neurons, while low-frequency stimulation of LA produces LTD of those synapses (Rammes et al., 2000; Azad et al., 2004).

The synapses onto ITCs of projection neurons in the basolateral amygdaloid complex (BLA), which includes LA and BA, undergo both LTP and LTD (Royer and Pare, 2002; Amano et al., 2010). LTP of those synapses can occur as a consequence of extinction training, but it appears to require input from IL because it does not occur if IL is inactivated (Amano et al., 2010). Projections from IL onto the ITCs appear to be important for extinction (Likhtik et al., 2008), but it is not known whether those synapses are modifiable. Fear conditioning can potentiate the synapse between BA and CEm (Amano et al., 2010), but it is not known whether the synapses from ITCm to CEm or the synapses between the ITCs (e.g., from ITCl to ITCm in Figure 1) are modifiable. Thus, most but not all of the synapses depicted in Figure 1 are known to undergo LTP and/or LTD. Changes in synaptic strength due to LTP or LTD are generally within 100% (i.e., LTP can increase synaptic strength by twice while LTD can decrease it to naught).

The cannabinoid receptor CB1 is highly expressed in several amygdaloid nuclei including LA and BA but excluding CE and the ITCs (McDonald and Mascagni, 2001). Within LA and BA, CB1 is found presynaptically on GABAergic interneuron terminals (Katona et al., 2001). During extinction training, CS presentation causes the endocannabinoid levels in LA and BA to rise (Marsicano et al., 2002). Apparently, the effect of endocannabinoid binding to CB1 in LA and BA is to cause LTD of the GABAergic synapses of interneurons onto projection neurons, because this LTD does not occur in CB1-deficient mice but it is enhanced in normal mice in which endocannabinoid degradation is blocked pharmacologically (Marsicano et al., 2002; Azad et al., 2004). Thus, LTD of GABAergic interneuron synapses onto LA and BA projection neurons is a concomitant of extinction, and both depend on cannabinoid signaling. The goal of the computational analysis is to find combinations of synaptic strength changes in amygdala that are compatible with both LTD of GABAergic interneuron synapses and extinction, but this presents a paradox as close examination of Figure 1 reveals.

A GABAergic interneuron synapse onto an LA projection neuron is depicted as the connection from LAi to LA in Figure 1. Similar GABAergic interneuron synapses that could occur onto BA projection neurons are not shown in Figure 1 for clarity. Although BA can inhibit CEm via ITCm, the net influence of LA and BA on CEm is excitatory (LA excites CEm over the BA excitatory pathway and over the ITC and CEl disinhibitory pathways). CB1-mediated LTD of GABAergic interneuron synapses onto LA and BA projection neurons is required for extinction (see above), but it would enhance LA and BA responses to CS and so increase, rather than decrease, the drive on CEm to produce CR (fear response) commands. This would seem to oppose, rather than promote, extinction, and that is the central paradox explored in this computational analysis, whose goal is to find combinations of synaptic strength changes in amygdala that are compatible with both LTD of GABAergic interneuron synapses and extinction.

### Computational analysis

The combinatorial search demanded by the goal of this analysis posed special challenges. They were met using a computational approach that differs in a crucial way from that taken in most modeling studies in neurobiology. Even after making necessary simplifying assumptions, typical studies explore only a tiny fraction of model parameter (including connection weight) spaces. Such an approach is useful for “proof of concept” studies, but would not be appropriate in the present context where the analytical goal is to determine which weight configurations are compatible with both LTD of GABAergic interneuron synapses and extinction, and which of those also conform to known neurobiological constraints. Any combinatorial search must avoid a combinatorial explosion, and the present analysis will proceed from simplified versions of the framework depicted in Figure 1. Rather than simplify arbitrarily, the analysis will focus on two established models of extinction that each represent different subsets of the connections illustrated in Figure 1 (see Results). Despite their differences the analysis will reveal a unifying synthesis concerning GABAergic LTD and extinction, and will also provide specific hypothesis by which the two different frameworks can each be tested experimentally.

The approach taken in this analysis is based on declarative programming. Declarative programming languages differ fundamentally from the more commonly known imperative programming languages. Whereas the structure of an imperative program dictates the order in which operations should be carried out, the declarations in a declarative program (also known as a specification) indicate what operations can occur without dictating their order. Using a declarative specification, applicable declarations can execute in any order. For the purposes of exploring the possible states of a system modeled using a declarative specification, applicable declarations can execute in *all* possible orders (within the limits of computational resources), and the entire tree of state transitions can be searched for states of interest (e.g., Huth and Ryan, 2004).

Declarative programming has been used for decades to model and analyze complex manmade systems, but its use in biology is quite recent (for reviews see Hlavacek et al., 2006; Fisher and Henzinger, 2007). The declarative programming language used here is called Maude (Clavel et al., 2007). Maude has been applied to general problems in biology (Eker et al., 2004; Talcott, 2008). Specific applications to neurobiology have recently appeared (Anastasio, 2011, 2013). This analysis is the first application of declarative programming in the emotional learning field.

In Maude a declaration is either an equation or a rule. An applicable equation must always execute, and in so doing it simplifies but does not change the state of the model system. In contrast, an applicable rule may execute or not, but by executing it changes the state of the model system. In the Maude specifications for both Model 1 and Model 2, rules produce allowed weight modifications while equations determine the effects of each weight change on model element responses. Thus, rule executions produce allowed connection weight changes and cause the model system to transition from one state to another. State-space search in Maude involves searching the state-transition tree, which Maude first constructs through rule executions as follows. From the initial state (depth *d* = 0), Maude executes every applicable rule. If *r* rules apply in the initial state, then there are *r* new states at depth *d* = 1. If *r* rules apply from any state at any depth, then there are *r*^d^ states at any depth *d*. The tree of state transitions widens rapidly until rule execution ceases. Maude then searches the state-transition tree for states of interest in a breadth-first manner. States of interest have explicit properties (e.g., LA = 1, BA = 2, etc.) and may be subject to certain conditions (e.g., such that CEm > 0). In this analysis almost all states of interest needed to satisfy certain conditions, which represented experimentally determined constraints.

The Maude specifications for Model 1 and Model 2 both terminate, meaning that rule executions in both do not proceed indefinitely but ultimately halt. All of the weight configurations reported for Model 1 and Model 2 correspond to terminal states. Model 1 and Model 2 were designed to produce extinction following fear conditioning. All of the weight configurations that achieve this without additional constraints, of which there were 19,273 for Model 1 and 8394 for Model 2 (see Results), can be found by an unconditional search for all terminal states. The configurations that produce extinction following fear conditioning with additional constraints were found by conditional searches. Each of the weight configurations reported resulted from a unique series of rule executions, but all series terminated before a tree depth of *d* = 20 was reached.

The structures of Model 1 and Model 2 were different but the procedures for making individual weight changes were the same in both. Generally, LTP occurred during simulated fear conditioning (i.e., excitatory or inhibitory weights could get more positive or negative, respectively), after which LTD occurred during simulated extinction (i.e., excitatory or inhibitory weights could get less positive or negative, respectively). In both models, individual weight changes were of absolute value 1 and all weights were bounded from 0 to 2, so that excitatory weights could only take values of 0, +1, or +2 while inhibitory weights could only take values of 0, −1, or −2. These levels correspond to the observed ranges of LTP and LTD, which can respectively double a synaptic strength or reduce it to 0 (see subsection on Amygdaloid plasticity). There is some uncertainty as to whether the endocannabinoid system facilitates extinction through associative or non-associative mechanisms (Kamprath et al., 2006). That and related issues are outside the focus of this analysis, which is concerned with the results rather than the mechanisms of plasticity. Thus, LTP and LTD are simply assumed to occur without taking into consideration the details of learning mechanisms.

Model 1 and Model 2 were also instantiated in MATLAB™, which is an imperative programming language widely used in neurobiology. The main reason for the MATLAB programs was to serve as crosschecks for the Maude specifications. The initial weight configuration and a battery of fear conditioned and extinguished weight configurations were checked for consistency between the programs written in the two different languages. A subsidiary reason for the MATLAB programs was to use them for directed searches to find real-valued sets of weight changes that would produce simulated extinction following simulated fear conditioning. These weight changes are constrained at the start and throughout a MATLAB directed search so that the weights, despite being changed by weight-change values, always remain within the absolute range of 0 to 2, which is the same range as for the integer weights in the Maude searches. All directed searches are initiated from random start points (i.e., sets of weight changes) that are uniformly distributed over the constrained space of weight changes.

A directed search in MATLAB proceeds by perturbing, in turn, each weight change by an amount Δ, keeping all other weight changes unperturbed, and finding the error associated with each new set of weight changes. The error for any set is the sum of the absolute differences between the desired and actual model outputs (i.e., PAG responses) in the unconditioned, conditioned, and extinguished cases. If a perturbed set has a lower error than the current (i.e., unperturbed) set, then the perturbed set with the lowest error becomes the new current set, Δ is doubled, and the perturbation/evaluation process is repeated. If no perturbed set has a lower error than the current set, then the current set is unchanged, Δ is halved, and the process is repeated. The whole procedure continues until Δ falls below 10^−6^. MATLAB was used to make 1000 searches for each of Model 1 and Model 2. For either model, the number of searches achieving zero error with LTD of inhibitory interneuron connections was expressed as a percentage of the number of searches achieving zero error with or without LTD of inhibitory interneuron connections. This percentage provided a gauge, using MATLAB directed search, on a random sample of real-valued weight configurations that was compared with an analogous percentage based on exhaustive search of integer-valued weight configurations using Maude. Comparison of the results of exhaustive, integer-valued search vs. random, real-valued search provides some assurance that the necessary restriction to integers for state-space searches did not limit the main findings (see Results).

Calculations were performed on a Pentium PC with dual, 2.8 GHz processors and with 2 GB of RAM. Programs were run under the Windows XP operating system. Maude calculations were performed using Core Maude version 2.4 as part of the Maude for Windows package, downloadable for free from the MOMENT website: moment.dsic.upc.es. The Maude search of integer space for all 19,273 of the terminal states for Model 1 required 4 min 52 s, while that for all 8394 terminal states for Model 2 required 2 min 24 s. For both models all constrained Maude searches required less time. Directed searches of continuous space were performed using MATLAB version R2010a. Each individual MATLAB directed search for Model 1 and Model 2 required 0.7 and 0.9 s, respectively (1000 searches required about 12 and 15 min, respectively). These methods are directly scalable to larger networks, subject to computational resources.

### Structure of model 1

**Figure 2:**
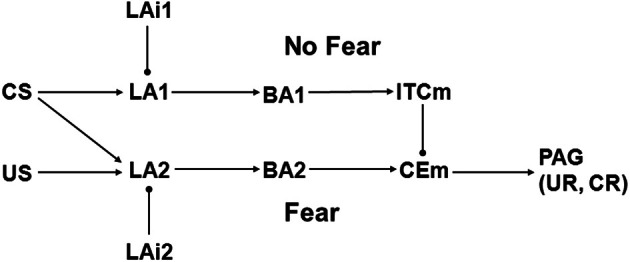
**Schematic illustrating Model 1, which is adapted from Lafenêtre, Chaouloff, and Marsicano (LCM) (Lafenetre et al., 2007).** BA1 and BA2 are excitatory projection neurons in BA, LA1, and LA2 are excitatory projection neurons in LA, and LAi1 and LAi2 are inhibitory interneurons in LA. Other abbreviations as in Figure 1. Biases are bBA1 = 0, bBA2 = 0, bCEm = 0, bITCm = 0, bLA1 = 2, bLA2 = 2, bLAi1 = 1, and bLAi2 = 1. Arrow and ball connections are positive and negative, respectively. All initial weights have absolute value 1 except those from CS to LA1 and LA2, which are 0.

**Figure 3:**
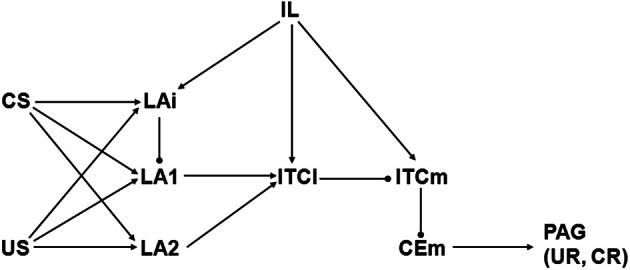
**Schematic illustrating Model 2, which is based on Paré, Quirk, and LeDoux (PQL) (Pare et al., 2004).** Abbreviations and symbols as in previous figures. Biases are bCEm = 1, bIL = 0, bITCl = −1, bITCm = 2, bLA1 = 2, bLA2 = 1, and bLAi = 1. All initial weights have absolute value 1 except those from CS to LAi, LA1, and LA2, and from IL to LAi, ITCl, and ITCm, which are initially 0.

Model 1 is based on a scheme proposed by Lafenêtre, Chaouloff, and Marsicano (LCM; Lafenetre et al., 2007), henceforth referred to as the LCM framework. A diagram of Model 1 is shown in Figure 2. The original LCM scheme was composed of four principle neurons and three interneurons in BLA, one neuron among the ITCs, and one neuron in CEm. These neural elements were meant to represent either single neurons or sets of neurons all having similar connectivity and behavior. The four BLA principle neurons were arranged into two parallel pathways, one labeled Fear and the other labeled No Fear. Model 1 departs from this scheme in two insignificant ways. First, the BLA is divided into the LA and the BA in Model 1, to maintain consistency with amygdaloid connectivity as described here (Figure 1) and with Model 2 (Figure 3). Second, only two rather than three inhibitory interneurons are represented in Model 1, one for each parallel pathway, because removal of the third interneuron, attached to the Fear pathway in the original scheme, affords a useful simplification but does not change model behavior in a way that alters the conclusions of the analysis. Otherwise, the structure of Model 1 is the same as that of the original LCM framework. In both, the Fear pathway directly excites CEm while the No Fear pathway indirectly inhibits it via ITCm. Note that Model 1, like the original LCM scheme, lacks input from IL. Both also exclude CEl, the role of which in extinction is still controversial (see subsection on Amygdaloid Connectivity).

As in LCM, the elements of Model 1 can be interpreted as single neurons or as groups of neurons all having the same connectivity and behavior. US and CS represent thalamic and cortical inputs to amygdala, while PAG drives UR and CR. The element labels also stand for the activity of each element, interpreted as the firing rate of each neuron (or group of neurons). For example, BA1 represents the firing rate of the BA neuron in the No Fear pathway. Connection weights, which represent synaptic strengths, are denoted descriptively. For example, wLA1toBA1 is the weight of the connection from LA1 to BA1 in the No Fear pathway. Initially, all connection weights have absolute value 1 except wCStoLA1 and wCStoLA2, which are initially 0. All connection weights in Model 1 are modifiable except wUStoLA2 and wCEmtoPAG. The modifiable weights in Model 1 can undergo LTP and LTD, which are increases and decreases in weight absolute values, respectively (see previous subsection). The most straightforward scheme for weight modification in Model 1 is for all modifiable weights to undergo LTP and LTD during fear conditioning and extinction, respectively, but many departures from this scheme will be required as part of Model 1 analysis.

The activity of any element in Model 1 is determined simply as the sum of its weighted inputs plus its bias. For example, the activity of CEm is computed as follows: CEm = bCEm + wBA2toCEm × BA2 + wITCmtoCEm × ITCm. Note that the CEm bias (bCEm) is 0 and wITCmtoCEm is negative. Except for CEm, all amygdaloid elements in Model 1 have initial spontaneous rates of 1. Note that US and CS are 0 under spontaneous conditions. The bias values for each element (listed in the Figure 2 caption) ensure that their initial spontaneous rates are 1, given the initial connection weights of absolute value 1 (except for wCStoLA1 and wCStoLA2, which are initially 0).

While GABAergic interneurons in amygdala are known to have much higher spontaneous rates than glutamatergic projection neurons (Bauer and Ledoux, 2004), the relative efficacies of excitatory vs. inhibitory synapses are not known. The analysis required that all connections have comparable efficacy because their weights were all restricted to the same absolute range. To ensure comparable connection efficacy, all model elements with non-zero spontaneous rate take the same initial rate of 1 (actually, any initial rate would serve this purpose as long as it is common to all the elements involved). To take an example, if BA2 and ITCm in Model 1 are both active at 1, and if their weights to CEm (wBA2toCEm and wITCmtoCEm) both increase in absolute terms from 1 to 2, then their effects on CEm still cancel. Such equivalence of efficacy is essential to Model 1 (and Model 2, see next subsection) and it is facilitated by having all amygdaloid elements (except CEm) express the same initial spontaneous rate.

In Model 1, CEm input to PAG greater than 0 activates PAG, which produces a UR or CR. In the initial state, switching US from 0 to 1 will activate the Fear pathway, and all of LA2, BA2, CEm, and PAG will be activated, resulting in UR. Model element activities change as a result of the connection weight adjustments made during simulated fear conditioning, and change again as a result of simulated extinction. Many of these Model 1 properties are the same for Model 2.

### Structure of model 2

Model 2 proceeds from a perspective offered by Paré, Quirk, and LeDoux (PQL; Pare et al., 2004), henceforth referred to as the PQL framework. A diagram of Model 2 is shown in Figure 3. The original PQL view represented neurons, or groups of neurons, in LA, ITCl, ITCm, CEm, and IL. Unlike LCM, PQL included IL but excluded BA. Like LCM, CEl is not included in the PQL view. Model 2 differs from the original PQL framework in two respects. First, LA interneurons were not represented in the original model but are represented in Model 2 (i.e., LAi). Second, US and CS inputs from thalamus and cortex projected directly to CEm as well as to LA in the original PQL view, but direct US and CS inputs are omitted from Model 2.

These changes have important consequences for model behavior but they had to be made in order to accomplish the goal of the analysis, which was to generate new hypotheses concerning the synaptic strength adjustments that underlie extinction. Because extinction requires cannabinoid signaling (Marsicano et al., 2002), and because CB1 receptors are located on inhibitory interneurons (Katona et al., 2001), these interneurons must be included in any model of extinction. Also, CEm lacks CB1 receptors (McDonald and Mascagni, 2001). Moreover, according to recent studies, neither fear conditioning nor extinction depends on synaptic plasticity within CEm proper (Ciocchi et al., 2010; Zimmerman and Maren, 2010). In contrast, lesions of LA prevent both fear conditioning and extinction (Ledoux et al., 1990; Calandreau et al., 2005). For these reasons, Model 2 depicts the pathway from US and CS through LA, including inhibitory LA interneurons, but it excludes direct projections from US and CS to CEm.

Naming conventions and model element activity computations are the same for Model 2 as for Model 1. As in Model 1, the weights of the connections from US (wUStoLAi, wUStoLA1, and wUStoLA2) and from CEm (wCEmtoPAG) are not modifiable. Unlike Model 1, wITCmtoCEm is not modifiable in Model 2, and neither is wITCltoITCm, because plasticity at those synapses has not been demonstrated experimentally (see subsection on Amygdaloid plasticity). All other connection weights in Model 2 are modifiable. As in Model 1, weight modification in Model 2 involves LTP and LTD, which are increases and decreases in weight absolute values, respectively. In Model 2, wCStoLAi, wCStoLA1, wCStoLA2, wLAitoLA1, wLA1toITCl, and wLA2toITCl can undergo LTP during fear conditioning and LTD during extinction. In contrast, wILtoLAi, wILtoITCl, and wILtoITCm can undergo LTP during extinction with no weight modification allowed during fear conditioning.

Initially, all connection weights in Model 2 have absolute value 1 except those from CS (wCStoLAi, wCStoLA1, and wCStoLA2) and those from IL (wILtoLAi, wILtoITCl, and wILtoITCm), which are initially 0. All elements in Model 2 except US, CS, CEm, PAG, and IL have non-zero initial spontaneous activity. Biases ensure that all non-zero initial spontaneous activities equal 1 (see caption to Figure 3). In the initial state, switching US from 0 to 1 results in activation of CEm. This activates PAG, which produces the UR.

In the Maude specifications for both Model 1 and Model 2, allowed weight modifications continue during fear conditioning until CS activity (CS = 1) alone can activate PAG. When fear conditioning has ended, different allowed weight modifications continue during extinction training until CS activity can no longer activate PAG. For both phases of learning, connection weights are modified one at a time, and every individual weight change is followed by evaluation of the effects of that weight change on the responses to CS of all neural elements. Weight modification terminates if PAG goes to 1 during fear conditioning or to 0 during extinction.

In Model 2, IL takes value 1 during extinction training but is otherwise 0. Note that Model 1 lacks element IL. Because the Maude specification exhaustively explores the model state space, extinction occurs from every possible weight configuration that supports fear conditioning in either model. These conditioned configurations include those in which the weighs of the connections of inhibitory interneurons onto LA projection neurons have undergone LTP. This is consistent with experiment because LTP of inhibitory interneuron synapses onto LA projection neurons can accompany the LTP of thalamic and cortical synapses onto LA projection neurons that is associated with fear conditioning (see subsection on Amygdaloid Plasticity). Because the focus is on extinction, weight configurations that support fear conditioning are not analyzed, but those that support extinction following fear conditioning are analyzed in detail.

## Results

The data on the effects of endocannabinoids on extinction present a puzzle. They indicate (see Introduction and Methods) that extinction is accompanied by LTD of the synapses onto LA projection neurons from inhibitory interneurons (LAi in Figure 1), but this would increase the responses of LA projection neurons, which would in turn increase the responses of the CEm neurons that command fear responses. Thus, it would seem that LTD of LAi to LA synapses would be inimical to extinction. Yet known amygdaloid organization offers many possible combinations of synaptic strength changes that could produce extinction of fear conditioning, and some of these may be compatible with LTD of LAi to LA synapses. In order to generate new hypotheses concerning the synaptic strength adjustments that underlie extinction, two different models of extinction are examined in detail. Model 1 (diagrammed in Figure 2) is based on the LCM scheme proposed by Lafenêtre, Chaouloff, and Marsicano (Lafenetre et al., 2007), while Model 2 (diagrammed in Figure 3) proceeds from the PQL perspective offered by Paré, Quirk, and LeDoux (Pare et al., 2004). Model 1 (based on LCM) and Model 2 (based on PQL) are described in detail in Methods.

### Analysis of model 1

The elements of Model 1 represent single neurons or groups of neurons either in amygdala, or that carry US or CS inputs to amygdala, or that receive UR or CR commands from amygdala (see Methods). As in the original LCM scheme, Model 1 is organized into Fear and No Fear pathways. In the Fear pathway, LA2 is excited by US, and can be excited by CS after fear conditioning. Then LA2 excites BA2, BA2 excites CEm, and CEm excites PAG, producing UR or CR. In the No Fear pathway, LA1 can be excited by CS or disinhibited by LAi1 after extinction training. Then LA1 excites BA1, BA1 excites ITCm, and ITCm inhibits CEm. Inhibition by ITCm can cancel excitation by BA2, thereby preventing the issue from CEm of commands to PAG for CR. Thus, Fear or No Fear behavior in Model 1 depends on the responses of its elements, and those depend on the weights of the connections between the elements. Model 1 was implemented by programs written in both Maude and MATLAB (see Methods), and a battery of identical weight adjustments were made in both. For all weight adjustments, model behavior was the same in both programs (not shown). While this does not prove that the Maude specification is free of bugs, it does reduce the odds that any of the results of the analysis have been corrupted by programming error.

Analysis of Model 1 begins by assuming that the weights of all connections are modifiable except wUStoLA2 and wCEmtoPAG. In that case there are 9 modifiable weights, and each weight has 3 possible values (0, +1, and +2 for excitatory connections; 0, −1, and −2 for inhibitory connections), so there are 3^9^ or 19,683 possible weight combinations. Of interest for the analysis are those combinations that produce extinction following fear conditioning, and especially those that produce extinction in conjunction with LTD of the weights of the connections of inhibitory interneurons LAi1 and LAi2 onto LA projection neurons LA1 and LA2, respectively. To determine whether wLAi1toLA1 or wLAi2toLA2 have undergone LTD during extinction training, their values immediately following fear condition must be recorded. This introduces two further weight parameters. Because each of wLAi1toLA1 and wLAi2toLA2 can either get more negative during fear conditioning (i.e., go to −2) or stay at their initial value (−1) each of the two weight-record parameters has two possible values, so the total number of possible weight (and weight-record) combinations increases by a factor of 2^2^ to 78,732.

In the original LCM scheme, extinction is thought to be mediated by potentiation of the No Fear pathway. This, in turn, is thought to occur through CB1-mediated LTD of the synapses of inhibitory interneurons onto the BLA projection neurons composing the No Fear pathway. That corresponds to LTD of wLAi1toLA1 in Model 1. Perhaps because LCM was designed to account for extinction in conjunction with LTD of inhibitory interneuron synapses, state-space search in Maude shows that many of the possible weight configurations are compatible with this condition. Of the total of 78,732 weight (including weight-record) configurations, 19,273 produce extinction following fear conditioning. Of those, 11,310 do so in conjunction with LTD of wLAi1toLA1.

In the analysis of Model 1 (summarized in Table 1), Maude exhaustively searches the entire state-space of weight configurations (see Methods). This is possible because the weights in the Maude specification are constrained to take only a limited number of integer values, but it raises the question of whether the restriction to integers is causing the analysis to miss configurations that rely on non-integer weights. There may be no definitive way to answer this question, but to get a rough idea the MATLAB version of Model 1 was used to make directed searches of real-valued weight configurations (see Methods). Out of 1000 directed searches, each starting from a random initial configuration, 560 produced extinction following fear conditioning, and of those 187 did so in conjunction with LTD of wLAi1toLA1. For comparison, about 59% of all configurations found by Maude integer-valued state-space search achieve extinction in conjunction with LTD of wLAi1toLA1, while only about 33% found by real-valued directed search in MATLAB do so. The real-valued directed search in MATLAB is not (nor could it be) exhaustive. It is merely a random sample of a real-valued space of weight configurations. The fact that the real-valued sample contained 26% fewer target configurations than the exhaustive, integer-valued search provides some assurance that the latter is not missing configurations that rely on non-integer weights.

**Table 1:** **Search conditions and numbers of compatible configurations for Model 1**.

**Conditions**	**Configurations**
(1) No conditions other than that the 9 modifiable weights are each restricted to three absolute levels (0, +1, +2 or 0, −1, −2) and all 9 can undergo LTP during fear conditioning and LTD during extinction	19,273
(2) 1 and LTD of wLAi1toLA1 (CB1-mediated LTD of wLAi1toLA1)	11,310
(3) 1 and LTD of wLAi2toLA2 (CB1-mediated LTD of wLAi2toLA2)	11,761
(4) 1 and LTD of wLAi1toLA1 and wLAi2toLA2 (i.e., 1 and 2 and 3)	6914
(5) 4 and wLAi1toLA1 equal to wLAi2toLA2 after extinction	3833
(6) 5 and wLAi1toLA1 also equal to wLAi2toLA2 after conditioning	2315
(7) 6 but with no plasticity of wITCmtoCEm	777
(8) 7 but with no conditioning of wCStoLA1 and with LTP and LTD allowed for wCStoLA1 and wCStoLA2, respectively, during extinction	773
(9) 8 but with LTP only (no LTD) allowed for both wCStoLA1 and wCStoLA2 during extinction (during conditioning, LTP still allowed for wCStoLA2 but no modification allowed for wCStoLA1)	619
(10) 9 but with all weight modification disallowed for wLA1toBA1, wBA1toITCm, wLA2toBA2, and wBA2toCEm	6
(11) 10 with wLAi1toLA1 and wLAi2toLA2 still equal before and after extinction but with LTD of wLAi1toLA1 and wLAi2toLA2 disallowed (CB1-mediated LTD cannot occur for either weight)	3
(12) 10 but with all weight modification disallowed for wCStoLA1	0
(13) 1 but with BA1 and BA2 removed	0

The results of searches of the state space of Model 1 are tabulated according to conditions and numbers of compatible weight configurations. The baseline conditions for all configurations are that they achieve extinction following fear conditioning and that the weights are limited to values of 0, +1, or +2 for excitatory weights and 0, −1, or −2 for inhibitory weights. The conditions become more restrictive with each subsequent row. For the least restrictive case (row 1), all 9 modifiable weights may undergo LTP during fear conditioning and LTD during extinction. Further restrictions involve additional limits on allowable weight values or weight changes. The state-space is searched after all allowed weight changes are made in all possible orders and combinations.

In the original LCM model, extinction is thought to occur through CB1-mediated LTD of the synapses of inhibitory interneurons onto the BLA projection neurons composing the No Fear pathway but not the Fear pathway, even though inhibitory interneurons contact the projection neurons of both pathways. In fact, in the original LCM model the Fear pathway has two inhibitory interneurons while the No Fear pathway has only one, but for present purposes the second inhibitory interneuron contacting the Fear pathway would be redundant and is not included in Model 1. Of direct concern is the idea that the CB1-mediated LTD of inhibitory interneuron synapses that occurs during extinction training would potentiate the No Fear pathway but would not potentiate the Fear pathway. This issue is of concern because BLA endocannabinoid levels rise during extinction training (Marsicano et al., 2002), and it is not clear why a general BLA endocannabinoid increase would cause CB1-mediated LTD of some inhibitory interneuron synapses but not others.

Model 1 was used to explore whether the restriction of CB1-mediated LTD to only the inhibitory interneuron synapses onto the BLA projection neurons of the No Fear pathway is actually a requirement of the LCM scheme. In Model 1, CB1-mediated LTD of the synapses of inhibitory interneurons onto the BLA projection neurons composing the Fear pathway corresponds to LTD of wLAi2toLA2. Of the 19,273 extinction configurations, 11,761 occur in conjunction with wLAi2toLA2 LTD. Moreover, 6914 occur in conjunction with LTD of both wLAi1toLA1 and wLAi2toLA2, 3833 occur in conjunction with LTD of both wLAi1toLA1 and wLAi2toLA2 and with wLAi1toLA1 equal to wLAi2toLA2 following extinction, and 2315 occur in conjunction with LTD of both wLAi1toLA1 and wLAi2toLA2 and with wLAi1toLA1 equal to wLAi2toLA2 both before and after extinction. Thus, extinction can be achieved in the LCM model even if CB1-mediated LTD of both pathways occurs. Restriction of CB1-mediated LTD to the synapses of inhibitory interneurons onto the BLA projection neurons of the No Fear pathway is unnecessary for the LCM framework.

The original LCM scheme focused on “potentiation” of the Fear or No Fear pathways, and this could be interpreted as strengthening of all of the synapses along either pathway. Indeed, there is evidence for plasticity of all of the synapses represented in the model, except for the one represented by wITCmtoCEm in Model 1 (see Methods). Disallowing modification of wITCmtoCEm reduces the total number of possible weight (and weight-record) configurations of Model 1 by a factor of 3 to 26,244. Of those, 777 weight configurations achieve extinction in conjunction with LTD of both wLAi1toLA1 and wLAi2toLA2 and with wLAi1toLA1 equal to wLAi2toLA2 both before and after extinction. Although plasticity of ITCm to CEm synapses may occur in the brain, its lack would not preclude extinction in conjunction with CB1-mediated LTD of inhibitory interneuron synapses, according to the LCM framework.

A larger issue concerns plasticity of the synapses of CS inputs onto BLA projection neurons in the LCM scheme. According to LCM (Lafenetre et al., 2007), only the BLA projection neurons of the Fear pathway receive both US and CS and only the Fear pathway could potentiate its response to CS during fear conditioning. Also according to LCM, only the No Fear pathway could potentiate its CS response during extinction, and only the Fear pathway could depotentiate its CS response during extinction. Up until now Model 1 was set up so that wCStoLA1 and wCStoLA2 could both undergo LTP and LTD during fear conditioning and extinction, respectively. To accord with the LCM scheme, Model 1 is altered to disallow any modification of wCStoLA1 during fear conditioning but to allow LTP of wCStoLA1 during extinction. LTP and LTD of wCStoLA2 during conditioning and extinction, respectively, is retained. These changes, which bring Model 1 in line with LCM in terms of CS to BLA synaptic plasticity, only reduces to 773 (from 777) the number of weight configurations that achieve extinction in conjunction with LTD of both wLAi1toLA1 and wLAi2toLA2 and with wLAi1toLA1 equal to wLAi2toLA2 both before and after extinction.

The LCM framework assumes that only the BLA projection neurons at the head of the Fear pathway (LA2 in Figure 2) receive both CS and US and that those BLA neurons project exclusively to other BLA neurons (BA2) that directly excite CEm (Lafenetre et al., 2007). LCM further assumes that only the BLA projection neurons at the head of the No Fear pathway (LA1 in Figure 2) receive only the CS and that those BLA neurons project exclusively to other BLA neurons (BA1) that indirectly inhibit CEm via ITCm. These assumptions are plausible but remain unproven (see subsection below on Experimental Tests). The Fear pathway depotentiation idea in LCM is based on findings that endocannabinoid release in amygdala reduces glutamatergic transmission (Azad et al., 2003). However, such release also reduces GABAergic transmission, and to the same extent as it reduces glutamatergic transmission (Azad et al., 2003). In any case, it is not clear why general endocannabinoid release during extinction training (Marsicano et al., 2002) would affect one amygdaloid pathway but not the other. The parsimonious assumption that endocannabinoid release affects both pathways equally, combined with the LCM assumption that CS potentiates the No Fear pathway during extinction, leads to the assumption that CS can also potentiate, or at least cannot depotentiate, the Fear pathway during extinction.

To address this issue Model 1 was again altered to disallow LTD but to allow LTP of wCStoLA2 during extinction. To summarize, Model 1 at this stage disallows any modification of wITCmtoCEm but it allows LTP and LTD of wLAi1toLA1 and wLAi2toLA2 during fear conditioning and extinction, respectively. It disallows modification of wCStoLA1 during conditioning but allows LTP of wCStoLA2 during conditioning and allows LTP of both wCStoLA1 and wCStoLA2 during extinction. Switching wCStoLA2 from possible LTD to possible LTP during extinction only reduces to 619 (from 773) the number of weight configurations that achieve extinction in conjunction with LTD of both wLAi1toLA1 and wLAi2toLA2 and with wLAi1toLA1 equal to wLAi2toLA2 before and after extinction. This number of compatible configurations depends not only on weight changes at the head of each pathway but also on weight changes in the middle and tail of each pathway. The original LCM framework does not seem compatible with this level of flexibility.

The LCM scheme plausibly assumes that potentiation (or depotentiation) can occur all along the two pathways, but changes occurring in the middle and tail of the pathways are secondary to changes occurring at the head. It is therefore of interest to explore the consequences of limiting weight changes to connections at the head of each pathway. Model 1 was again altered to disallow all changes to the downstream pathway weights wLA1toBA1, wBA1toITCm, wLA2toBA2, and wBA2toCEm. Only the following weight changes are still allowed: LTP of wCStoLA2 during fear conditioning, LTP of both wCStoLA1 and wCStoLA2 during extinction, LTP of both wLAi1toLA1 and wLAi2toLA2 during conditioning, and LTD of both wLAi1toLA1 and wLAi2toLA2 during extinction. At this stage there are only 4 modifiable weights in Model 1, reducing the total number of weight (and weight-parameter) configurations to 3^4^ × 2^2^ or 324. Of these only 6 configurations achieve extinction in conjunction with LTD of both wLAi1toLA1 and wLAi2toLA2 and with wLAi1toLA1 equal to wLAi2toLA2 both before and after extinction.

In all 6 cases, both LA1 and LA2 respond to CS following extinction. This is consistent with findings that some neurons in LA respond to CS after extinction training (Repa et al., 2001; Hobin et al., 2003). Also in all 6 cases, LA1 and LA2 respond equally to CS because wCStoLA1 equals wCStoLA2, and wLAi1toLA1 equals wLAi2toLA2. Furthermore, all of the elements in each of their pathways have the same response, so that LA1, LA2, BA1, BA2, and ITCm all respond equally to CS following extinction. This is consistent with the idea of whole-pathway potentiation in the original LCM scheme (Lafenetre et al., 2007). The equal responding of both the Fear and No Fear pathways following extinction is a consequence of whole-pathway potentiation, and of disallowing LTD of wCStoLA2 during extinction. Because BA1 and BA2 respond equally to CS following extinction, Model 1 does not reproduce the results of Herry and coworkers (Herry et al., 2008), who found some neurons in BA that respond to CS after fear conditioning but not after extinction. This discrepancy suggests that LTD of the Fear pathway may indeed occur in amygdala during extinction training, but it is not clear at present how that could happen within the LCM framework (see above in this subsection).

The analysis of Model 1 reveals that the LCM scheme (Lafenetre et al., 2007) is robust. It is able to demonstrate extinction in conjunction with CB1-mediated LTD of inhibitory interneuron synapses, even given the parsimonious assumption that this LTD must occur equally for all such synapses, and given the weight modification restrictions implied by whole-pathway potentiation. Because CB1-mediated LTD of inhibitory interneuron synapses is of central concern, it is of interest to explore the consequences of eliminating it in Model 1. If LTD of wLAi1toLA1 and wLAi2toLA2 during extinction is *disallowed* but all other restrictions made up to this stage are retained, then the number of configurations that achieve extinction with wLAi1toLA1 equal to wLAi2toLA2 both before and after extinction drops to 3. This result shows that LTD of inhibitory interneuron synapses increases (in fact, doubles) the number of configurations that achieve extinction in Model 1, and implies that CB1-mediated LTD of inhibitory interneuron synapses plays an important role in extinction (see Discussion).

The ability of the LCM framework to account for extinction in conjunction with CB1-mediated LTD of inhibitory interneuron synapses is due to its balanced architecture, in which the CEm response depends on the relative activity of the Fear and No Fear pathways. This can be demonstrated by disallowing modification of the CS input to the No Fear pathway, but again allowing LTD of wLAi1toLA1 and wLAi2toLA2 during extinction. Now only the following weight changes are allowed: LTP of wCStoLA2 during fear conditioning and extinction (no modification of wCStoLA1 is allowed during conditioning or extinction), LTP of both wLAi1toLA1 and wLAi2toLA2 during conditioning, and LTD of both wLAi1toLA1 and wLAi2toLA2 during extinction. Now there are only 3 modifiable weights in Model 1, reducing the total number of weight (and weight-parameter) configurations to 3^3^ × 2^2^ or 108. Of these the number of configurations that achieve extinction in conjunction with LTD of both wLAi1toLA1 and wLAi2toLA2 and with wLAi1toLA1 equal to wLAi2toLA2 both before and after extinction is 0.

This result reveals that the parallel Fear and No Fear architecture is essential to the LCM scheme. Weight adjustments accomplish fear conditioning when CS responding is greater in the Fear than in the No Fear pathway, and accomplish extinction when CS responding in the No Fear pathway increases enough to balance that in the Fear pathway. Given the progressively more restrictive constraints applied in Model 1 analysis, balancing the Fear and No Fear pathways required that wCStoLA1 equaled wCStoLA2 and that wLAi1toLA1 equaled wLAi2toLA2 after extinction. Achievement of this Fear and No Fear balancing requires LTP of the synapses of CS inputs onto some LA neurons during extinction training, which could be verified experimentally. Yet Model 1 analysis reveals that the feature of the LCM framework that is critical for it to accomplish extinction in conjunction with GABAergic LTD is the existence of parallel Fear and No Fear pathways. Model 1 analysis also shows that certain LCM assumptions, including that CB1-mediated LTD affects some GABAergic synapses but not others, are not critical to it. The analysis suggests that experimental verification of Fear and No Fear pathway connectivity would be the most effective way to test the validity of the LCM framework (see subsection below on Experimental Tests).

The LCM architecture hinges on the fact that BA can both excite CEm directly and inhibit CEm indirectly via ITCm (see Methods). However, lesion studies call into question the idea that BA is essential for all forms of fear conditioning and extinction. Although BA lesions disrupt context conditioning (Calandreau et al., 2005; Onishi and Xavier, 2010), and BA lesions made after cue conditioning prevent the expression of conditioned fear (Anglada-Figueroa and Quirk, 2005), BA lesions made before cue conditioning have no effect on fear conditioning to a cue or its extinction (Nader et al., 2001; Sotres-Bayon et al., 2004; Anglada-Figueroa and Quirk, 2005; Calandreau et al., 2005). Because the models analyzed here are based on data derived from cue conditioning studies, it is of interest to explore weight configurations in models that exclude BA. It is obvious that removal of BA1 and BA2 from Model 1 will prevent UR and CR under all circumstances and fear conditioning should be impossible. To test this robustly, Model 1 was first returned to full connection weight modifiability. As expected, with BA1 and BA2 removed from Model 1, the number of weight configurations that are compatible with extinction following fear conditioning is 0. This obvious result in no way impugns the LCM framework, which was designed to take the role of BA into account, but it does raise the question of how the endocannabinoid system could contribute to extinction in a model that excludes BA. We now turn to such a model.

### Analysis of model 2

Model 2 adheres to the PQL view that US or, after conditioning, CS input to amygdala excites neurons in LA, and that LA indirectly activates amygdala output neurons in CEm that command PAG to drive UR or CR. The PQL framework promotes the idea that LA activates CEm by exciting ITCl, which inhibits ITCm, thereby releasing CEm from ITCm inhibition. Thus, fear conditioning and extinction in Model 2 must involve increased and decreased LA influence, respectively. Therein lays a conundrum.

Extinction requires activation by endocannabinoids of CB1 receptors, which leads to LTD of the synapses of inhibitory interneurons onto BLA (including LA) projection neurons (Marsicano et al., 2002), but such LTD would increase LA responding to CS, which would oppose extinction. Model 1 (based on LCM), solved this problem in part by having separate Fear and No Fear pathways. LTD of inhibitory interneuron synapses onto BLA projection neurons would likely affect both pathways, so in the two-pathway framework its effects can at least balance out, leaving extinction in Model 1 to the modification of other connection weights. This problem is faced full on in Model 2 (based on PQL), which has essentially a single Fear pathway, and the main object of Model 2 analysis is to find weight configurations that achieve extinction in conjunction with LTD of the synapses of inhibitory interneurons onto LA projection neurons.

Model 2 depicts three LA neurons: LA1 and LA2 are projection neurons, and LAi is an inhibitory interneuron. All of these LA neurons receive both US and CS input. Not all LA projection neurons are thought to receive inhibitory interneuron input (Rosenkranz and Grace, 2002; Likhtik et al., 2005), so LAi contacts LA1 but not LA2 in Model 2. Because endocannabinoids produce LTD of the synapses of inhibitory interneurons onto projection neurons in BLA (Marsicano et al., 2002), the connection from LAi to LA1 is of particular importance in Model 2 analysis. IL contacts inhibitory interneurons in LA as well as the ITCs (see Methods), so IL contacts LAi, ITCl, and ITCm in Model 2.

As for Model 1, Model 2 was implemented by programs written in both Maude and MATLAB (see Methods), and a battery of identical weight adjustments were made in both. For all weight adjustments, model behavior was the same in both programs (not shown). As for Model 1, this crosscheck strengthens confidence that the results of analysis of Model 2 have not been corrupted by programming error.

Analysis of Model 2 begins by assuming that the weights of all connections are modifiable except those from US (wUStoLAi, wUStoLA1, and wUStoLA2), from the ITCs (wITCltoITCm and wITCmtoCEm), and from CEm (wCEmtoPAG). Therefore, as has Model 1, Model 2 has 9 modifiable weights, and each weight has 3 possible values (0, +1, +2, or 0, −1, −2), so there are 3^9^ or 19,683 possible weight combinations. Of critical importance for the analysis is to search for weight combinations (configurations) that achieve extinction in conjunction with LTD of the weight of the connection from LAi to LA1. For the purpose of determining whether wLAitoLA1 has undergone LTD during extinction training, an additional weight-record parameter is added to record its value immediately following fear conditioning. Because wLAitoLA1 can either get more negative during fear conditioning (i.e., go to –2) or stay at its initial value (–1), the weight-record parameter has 2 possible values, so the total number of possible weight (and weight-record) combinations increases by a factor of 2 to 39,366.

Of the total of 39,366 weight (including weight-record) configurations in Model 2, 8394 produce extinction following fear conditioning. Of those, 4659 do so in conjunction with LTD of wLAitoLA1. These and other analysis results for Model 2 (summarized in Table 2) are produced using Maude integer-valued state-space searches. As for Model 1, the MATLAB version of Model 2 was also used to make real-valued directed searches from random start states (see Methods). Out of 1000 directed searches, 743 produced extinction following fear conditioning, and of those 113 did so in conjunction with LTD of wLAitoLA1. For comparison, about 56% of all configurations found by Maude integer state-space search achieve extinction in conjunction with LTD of wLAitoLA1, while only 18% of all configurations found by real directed search in MATLAB do so. The fact that the real-valued random sample contained 38% fewer target configurations than the exhaustive, integer-valued search provides some assurance that the latter is not missing configurations that rely on non-integer weights.

**Table 2:** **Search conditions and numbers of compatible configurations for Model 2**.

**Conditions**	**Configurations**
(1) No conditions other than that the 9 modifiable weights are each restricted to three absolute levels (0, +1, +2 or 0, −1, −2) and the 3 weights from IL are limited to LTP during extinction but the other 6 can undergo LTP during fear conditioning and LTD during extinction	8394
(2) 1 and LTD of wLAitoLA1 (CB-1 mediated LTD must occur)	4659
(3) 2 but with LA1 or LA2 excited by CS after extinction	2335
(4) 3 but with neither LA1 nor LA2 inhibited by CS after extinction	957
(5) 4 but with no LTD of wLA1toITCl or wLA2toITCl	129
(6) 5 but with no modification of wILtoLAi, wILtoITCl, or wILtoITCm	0
(7) 6 but with LTD of wLA1toITCl or wLA2toITCl allowed (again no modification of wILtoLAi, wILtoITCl, or wILtoITCm is allowed)	49
(8) 4 with weights constrained to be equal from CS (wCStoLAi = wCStoLA1 = wCStoLA2), from LA (wLA1toITCl = wLA2toITCl), and from IL (wILtoLAi = wILtoITCl = wILtoITCm)	0
(9) 8 with equality constraints as listed but with wLAitoLA1 free not to undergo LTD during extinction (it could do so or stay the same)	0
(10) 4 with weights from CS (wCStoLAi, wCStoLA1, and wCStoLA2) free to be unequal but with these equality constraints: (wLA1toITCl = wLA2toITCl) and (wILtoLAi = wILtoITCl = wILtoITCm)	0
(11) 4 with weights from LA (wLA1toITCl and wLA2toITCl) free to be unequal but with these equality constraints: (wCStoLAi = wCStoLA1 = wCStoLA2) and (wILtoLAi = wILtoITCl = wILtoITCm)	21
(12) 4 with weights from IL (wILtoLAi, wILtoITCl, and wILtoITCm) free to be unequal but with these equality constraints: (wCStoLAi = wCStoLA1 = wCStoLA2) and (wLA1toITCl = wLA2toITCl)	42
(13) 4 (with no equality constraints) but with LTD of wLAitoLA1 disallowed (CB-1 mediated LTD cannot occur)	303

The results of searches of the state space of Model 2 are tabulated according to conditions and numbers of compatible weight configurations. The procedures are largely the same as for Table 1 except that conditions do not necessarily become more restrictive with each subsequent row, and further restrictions can involve constraints on model element responses as well as on weight values or allowed weight changes.

Although Model 2 does not have explicit Fear and No Fear pathways, it does have two pathways through LA to ITCl. LA1 and LA2 both project to ITCl, and the relative activity of LA1 and LA2 is important for Model 2 behavior. Some but not all real LA neurons continue to respond to CS after extinction (Repa et al., 2001; Hobin et al., 2003), so at least one of LA1 and LA2 should have a CS response after simulated extinction in Model 2. Of the 4659 configurations that achieve extinction in conjunction with LTD of wLAitoLA1, 2335 have either LA1 or LA2 responding to CS. While some real BA neurons that were excited by CS following conditioning were actually inhibited by CS after extinction, neurons that were inhibited by CS after extinction were expressly searched for but not observed in LA (Herry et al., 2008). The implication of this data for Model 2 is that LA1 or LA2 should be excited by CS following extinction, but neither LA1 nor LA2 should be inhibited by CS following extinction (note that CS cannot inhibit LA2 but CS can cause net inhibition of LA1 via LAi). The number of configurations that have this property and also achieve extinction in conjunction with LTD of wLAitoLA1 is 957. This particular result will become important again at the end of Model 2 analysis.

With either LA1 or LA2 excited by CS following extinction training and neither of them inhibited by CS, some CS drive from LA remains after simulated extinction in Model 2. This suggests that extinction is mediated in part by connection weight changes downstream of LA. Because wITCltoITCm, wITCmtoCEm, and wCEmtoPAG are not modifiable in Model 2, and because LA1 and LA2 converge on and excite ITCl, it would seem that LTD of the connections from LA to ITCl would play an important role in simulated extinction in Model 2. Indeed, with either LA1 or LA2 excited but neither inhibited by CS after extinction, and with LTD of neither wLA1toITCl nor wLA2toITCl, only 129 configurations achieve extinction in conjunction with LTD of wLAitoLA1.

With LTD of neither wLA1toITCl nor wLA2toITCl, the weight changes mediating extinction in Model 2 must involve those of the connections from IL. Indeed, if all of the IL connection weights (wILtoLAi, wILtoITCl, and wILtoITCm) are constrained to be 0, and with LTD of neither wLA1toITCl nor wLA2toITCl, and with either LA1 or LA2 excited but neither inhibited by CS after extinction, then the number of Model 2 configurations that achieve extinction in conjunction with LTD of wLAitoLA1 is 0. However, if all the IL connection weights (wILtoLAi, wILtoITCl, and wILtoITCm) are constrained to be 0, but the LA to ITCl weights (wLA1toITCl and wLA2toITCl) are free to undergo LTD during extinction training, then the number of Model 2 configurations that achieve extinction with either LA1 or LA2 excited but neither inhibited by CS and in conjunction with LTD of wLAitoLA1 rises to 49.

These results can be taken as a prediction of Model 2. Given experimental findings that extinction occurs in conjunction with LTD of the synapses of inhibitory interneurons onto LA projection neurons, and that some LA projection neurons are excited by CS after extinction but none are inhibited, then Model 2 predicts that extinction must be associated with LTD of the synapses of LA neurons onto ITCl, or modification of the synapses of IL neurons onto inhibitory neurons in LA and ITC. This prediction comes with the caveat that Model 2 excludes BA. It also comes with the proviso that the weights of the connections from IL can be modified independently of one another (see below in this subsection). Whether or not they can be is a matter of conjecture, and it points up another way in which Model 2 differs from Model 1.

Model 2 also differs from Model 1 in that it does not take a whole-pathway approach but, instead, assumes that the responses of elements along the pathway from LA to ITCl, and from ITCl to ITCm, can be modified by input from IL. Therein lays another conundrum. IL activates LAi, ITCl, and ITCm, and it promotes extinction by activating LAi or ITCm but it promotes the opposite (fear responding) by activating ITCl. It appears that the pattern of IL influence should be of critical importance to extinction in Model 2.

Model 2 assumes that the synapses of IL neurons onto amygdala neurons are plastic, but it is not known whether they are (see Methods), much less whether or not they can be differentially modified. For that matter, it is not known whether synapses of CS inputs onto LA neurons, or of LA neurons onto ITCl, can be differentially modified. The most parsimonious assumption is that all synapses onto downstream neurons from the same subregion are plastically modified by the same amount, but making this assumption has serious consequences for Model 2. If the weights of the connections from CS are constrained to be equal (wCStoLAi = wCStoLA1 = wCStoLA2), and if the weights from LA projection neurons are constrained to be equal (wLA1toITCl = wLA2toITCl), and if the weights from IL are constrained to be equal (wILtoLAi = wILtoITCl = wILtoITCm), and if either LA1 or LA2 are excited but neither are inhibited by CS, then the number of configurations that achieve extinction in conjunction with LTD of wLAitoLA1 is 0. Interestingly, the requirement for LTD of wLAitoLA1 is not the culprit here because, given the same equality constraints and restrictions on LA1 and LA2 activity, the number of configurations that achieve extinction with or without LTD of wLAitoLA1 is again 0.

These results show that if the weights of the connections from CS are constrained to be equal (wCStoLAi = wCStoLA1 = wCStoLA2), and if the weights from LA projection neurons are constrained to be equal (wLA1toITCl = wLA2toITCl), and if the weights from IL are constrained to be equal (wILtoLAi = wILtoITCl = wILtoITCm), and if either LA1 or LA2 are excited but neither are inhibited by CS, then Model 2 cannot achieve extinction in conjunction with LTD of wLAitoLA1. If the connection weights from CS can be differentially modified but not those from LA and IL, then Model 2 again cannot achieve extinction in conjunction with LTD of wLAitoLA1 and either LA1 or LA2 excited but neither inhibited by CS. However, if the connection weights from LA alone, or if those from IL alone, can be differentially modified, with the others (from CS and IL, or from CS and LA, respectively) constrained to be equal, then there are 21 and 42 configurations, respectively, that achieve extinction in conjunction with LTD of wLAitoLA1 and either LA1 or LA2 excited but neither inhibited by CS. Thus, Model 2 predicts that, in preparations in which BA has been lesioned, extinction in conjunction with LTD of the synapses of inhibitory interneurons onto LA projection neurons can occur only if the synapses of LA projection neurons onto ITCl, or those of IL neurons onto LA inhibitory interneurons, ITCl, and ITCm, are not only plastic but differentially modifiable.

Differential modifiability means that plastic changes in synapses onto ITCl of LA projection neurons that receive input from inhibitory interneurons can be different from those of LA projection neurons that do not receive such input. It also means that changes in synapses of IL neurons onto ITCl can be different from those onto ITCm or onto LA inhibitory interneurons. Model 2 analysis suggests that the answer to the conundrum of IL innervation is that synapses of IL neurons onto LA inhibitory interneurons, ITCl, and ITCm can all change together by equal amounts, but only if synapses of LA projection neurons onto ITCl can change by unequal amounts. The reverse is also true: synapses of LA projection neurons onto ITCl can change together by equal amounts but only if synapses of IL neurons onto LA inhibitory interneurons, ITCl, and ITCm can change by unequal amounts.

While the results may go some way toward solving the puzzle of IL innervation, the question remains of how CB1-mediated LTD of the synapses of LA inhibitory interneurons onto LA projection neurons actually contributes to extinction, since such LTD would seem to oppose extinction. This issue is approached using one more search. To make it robust, all equality constraints are dropped (so weights of connections from CS, LA, and IL can all be differentially modified again). Then LTD of wLAitoLA1 during simulated extinction is *disallowed* in Model 2. This would correspond to elimination of CB1-mediated LTD in amygdala. With connection weights from CS, LA, and IL all differentially modifiable, and either LA1 or LA2 excited but neither inhibited by CS after extinction, the number of configurations that achieve extinction *without* LTD of wLAitoLA1 is 303. For direct comparison, a previous search reveals that, with connection weights from CS, LA, and IL all differentially modifiable, and either LA1 or LA2 excited but neither inhibited by CS after extinction, the number of configurations that achieve extinction *with* LTD of wLAitoLA1 is 957. These results show that LTD of inhibitory interneuron synapses is not only compatible with extinction, but it actually increases (in fact, triples) the number of weight configurations that achieve extinction while preserving some LA responses to CS in Model 2 (see Discussion).

### Experimental tests

Experiments to test the essential features of the LCM and PQL frameworks could employ techniques similar to those that have provided the current body of data on amygdaloid connectivity and plasticity (see Methods), but these would have to be augmented with techniques for accurate assessment of connectivity such as those based on spike-triggered averaging between pairs of extra- and intra-cellularly recorded neurons (Hempel et al., 2002) or statistical analysis of multiple neuron spike trains (Chen et al., 2011). Testing the features that are critical for the LCM framework to account for extinction in conjunction with GABAergic LTD would involve demonstrating the Fear and No Fear pathways. Showing that at least some CEm neurons receive excitatory input from BA neurons, that those BA neurons receive excitatory input from LA neurons, and that those LA neurons can develop CS responses during fear conditioning would provide evidence for the Fear pathway. Similarly, showing that at least some ITCm neurons receive excitatory input from BA neurons, that those BA neurons receive excitatory input from LA neurons, and that those LA neurons develop CS responses during extinction would provide evidence for the No Fear pathway.

Testing the features that are critical for the PQL framework to account for extinction in conjunction with GABAergic LTD would involve demonstrating the differential modifiability of LA or IL neuron synapses onto postsynaptic neurons, in the absence of influence from BA. Showing that the synapses onto ITCl neurons of at least some LA neurons that receive inhibitory interneuron input undergo LTD during extinction, while those of some other LA neurons that do not receive inhibitory interneuron input do not undergo LTD during extinction, would provide evidence of differential modifiability. Similarly, showing that some synapses from IL onto ITCm undergo LTP while others from IL onto ITCl do not undergo LTP during extinction would also provide evidence of differential modifiability. However, the analysis shows that the weight configurations that are consistent with extinction, along with GABAergic LTD and some preserved LA responding to CS, also encompass a wide range of configurations of the strengths of synapses of LA and IL neurons onto postsynaptic neurons, so the specific differences outlined here are examples of the many synaptic strength change differences that would provide support for the differential modifiability that is critical to the PQL framework.

## Discussion

The models analyzed in this study are based on a rich dataset, but the main question addressed in their analysis was prompted by the seminal work of Marsicano et al. (2002) who showed that extinction of fear conditioning requires activation of endocannabinoid CB1 receptors, and that such activation causes LTD of the GABAergic synapses of inhibitory interneurons onto BLA projection neurons. BLA includes LA, LA drives CEm to command conditioned fear responses (Ledoux et al., 1990; Royer et al., 1999; Ledoux, 2000), and CB1-mediated LTD of inhibitory interneuron synapses onto LA projection neurons could only enhance projection neuron activity and so enhance fear responses, which would opposes extinction. The main question, then, is how CB1-mediated inhibitory interneuron LTD could even be compatible with, much less be required for, extinction of fear conditioning.

Model 1 (based on the LCM scheme; Lafenetre et al., 2007) and Model 2 (based on the PQL view; Pare et al., 2004) provided two different structures within which to search out the compatibility question, and in both cases the key to a potential answer was the constraint, imposed by experimental findings, that some real LA neurons continue to respond to CS after extinction but none are inhibited by it (Repa et al., 2001; Hobin et al., 2003; Herry et al., 2008). Given this constraint, state-space search revealed that LTD of inhibitory interneuron synapses onto LA projection neurons caused the number of connection weight configurations that achieved extinction following fear conditioning to double for Model 1 and to triple for Model 2 (see Results). Numerous lines of evidence suggest that extinction of fear conditioning is a form of new, inhibitory learning rather than forgetting (Myers and Davis, 2007). By contributing to the maintenance of CS responses in LA after extinction, CB1-mediated inhibitory interneuron LTD could help to preserve fear memory in amygdala even after the amygdala has learned to inhibit responses to a formerly conditioned cue.

The main finding of the analyses is that CB1-mediated LTD of the GABAergic synapses of LA inhibitory interneurons onto LA projection neurons is not only compatible with extinction, but it increases the number of connection weight configurations that achieve extinction and also preserve some fear memory in amygdala, given model structures and other constraints based on experimental findings. By showing that the number of such configurations is increased twofold for Model 1 and threefold for Model 2, the analysis identifies preservation of fear memory during extinction as a highly likely function of CB1-mediated GABAergic LTD.

The increases in numbers of model weight configurations are important for two reasons. The first concerns the relative lack of knowledge concerning mechanisms of neural plasticity in amygdala, compared with the relative abundance of knowledge concerning amygdaloid anatomy and physiology. We know that the amygdala brings about fear conditioning and extinction by making synaptic strength changes but, because we are still unsure about how these synaptic strength changes are made, we could justifiably assume that, given well-established models based on known amygdaloid connectivity, the outcome associated with the most numerous synaptic strength configurations is the one most likely to occur in the real amygdala. Because CB1-mediated GABAergic LTD doubles in Model 1, and triples in Model 2, the number of weight configurations that achieve extinction along with preservation of fear memory in those well-established models, we can conclude that preservation of fear memory in amygdala during extinction is a highly probable function of CB1-mediated GABAergic LTD.

The second reason that increases in numbers of weight configurations are important is that the actual plastic mechanisms that bring about fear conditioning and extinction may have substantial random components. Two recent models that suggest possible adaptive mechanisms employ reinforcement signals and/or stochasticity (Krasne et al., 2011; Vlachos et al., 2011). Artificial neural networks trained using various learning algorithms that have a stochastic (random) component, including those using reinforcement signals, share the property that the number of iterations required to train them can decrease with increases (within limits) in the number of ways the network has to solve the learning problem (Anastasio, 2010). LTD of the synapses of inhibitory interneurons onto LA projection neurons increases the number of configurations that are compatible with extinction and some retained CS memory in both Model 1 and Model 2. It is possible that CB1-mediated GABAergic LTD in the real LA increases the amygdala's options for adaptation sufficiently for extinction with some retention of CS memory to occur.

In both Model 1 and Model 2, elements could be interpreted either as single neurons or as groups of neurons all having the same connectivity and behavior. While the former interpretation suggests how individual neurons might respond, the latter interpretation suggests how extinction of fear conditioning might result from changes in the strengths of the influences that various cortical and amygdaloid subregions have on one another. Obviously, brain subregions are composed of many more than one or two neurons. Increasing the number of model elements in each subregion would geometrically increase the number of weight configurations that produce any desired outcome, but would not change the relative numbers of those that produce different outcomes if element numbers were increased uniformly. Specifically, uniformly increasing the number of each element in Model 1 or Model 2 would increase the number of weight configurations that produce any particular outcome, but the configurations that achieve extinction and some fear memory preservation with LTD of inhibitory interneuron synapses would still outnumber those that do so without it.

An important distinction between the two models is that retention of CS responses in LA emerges automatically in Model 1, as a consequence of its balanced architecture and parsimony assumptions, while retention of CS responses in LA pertain only for a subset of compatible configurations in Model 2. Considering the differences in the LCM and PQL schemes, and so in Model 1 and Model 2, it is remarkable that CB1-mediated LTD of BLA inhibitory interneuron synapses actually increases the options that both Model 1 and Model 2 have available to learn to inhibit a previously conditioned fear response and to preserve some LA responding to CS. Both models, and the LCM and PQL frameworks on which they are based, depend sensitively on specific patterns of connectivity and plasticity, but both also show how extinction of fear conditioning requires an interaction between several amygdaloid subregions and cortical and thalamic inputs to the amygdala.

The LCM and PQL frameworks (Pare et al., 2004; Lafenetre et al., 2007) were chosen for analysis because they are well-established, represent different but well-justified simplifications of amygdaloid connectivity, and include only known connections. The analysis of the LCM and PQL frameworks conducted here shows that both are compatible with extinction in conjunction with inhibitory interneuron LTD and preserved fear responding in amygdala, and this consistency with observation reinforces the plausibility of both frameworks. A very recent framework (Pare and Duvarci, 2012), which includes BA and CEl and also includes hypothetical as well as known connections, has a much larger state (weight configuration) space than the models analyzed here. If the hypothetical connections proposed in that new framework are confirmed, then it would be of interest to explore its state space using the computational techniques introduced here. For this initial foray, however, it was beneficial to focus on established and essential connections in order to derive specific and testable predictions (see Results).

The computational approach taken here is fundamentally different from that taken in typical modeling studies in neurobiology, which employ imperative programming and are limited to simulation. This approach is based on declarative programming, which readily offers options in addition to simulation such as analysis of model behavior using temporal logic and state-space search (Huth and Ryan, 2004). Here the Maude specifications of Model 1 and Model 2 were used to exhaustively search model state spaces for weight configurations that satisfied specific sets of constraints. The models are relatively simple, but the numbers of their possible weight (and weight parameter) configurations run into the tens of thousands—obviously too many to evaluate through separate simulations. The state-space searches provided definitive answers concerning model behavior. They showed that both models can use CB1-mediated LTD of inhibitory interneuron synapses to achieve extinction of fear conditioning while preserving fear memory, but only if well-defined and experimentally verifiable conditions of amygdaloid connectivity and plasticity are met. The analysis also provides a concrete example of how declarative programming and its associated tools can be applied to other complex problems in emotional learning and in neurobiology more generally.

### Conflict of interest statement

The author declares that the research was conducted in the absence of any commercial or financial relationships that could be construed as a potential conflict of interest.
